# Coral metabolite gradients affect microbial community structures and act as a disease cue

**DOI:** 10.1038/s42003-018-0189-1

**Published:** 2018-11-05

**Authors:** Michael A. Ochsenkühn, Philippe Schmitt-Kopplin, Mourad Harir, Shady A. Amin

**Affiliations:** 1 Biology Division, New York University Abu Dhabi, Saadiyat Island, Abu Dhabi, 129188 United Arab Emirates; 2 Research Unit Analytical BioGeoChemistry, Helmholtz Centre Munich, Ingolstädter Landstraße 1, 85764 Neuherberg, Germany; 3 Lehrstuhl für Analytische Lebensmittelchemie, Technische Universität München, Alte Akademie 10, 85354 Freising, Germany; 4 Chemistry Division, New York University Abu Dhabi, Saadiyat Island, Abu Dhabi, 129188 United Arab Emirates

## Abstract

Corals are threatened worldwide due to prevalence of disease and bleaching. Recent studies suggest the ability of corals to resist disease is dependent on maintaining healthy microbiomes that span coral tissues and surfaces, the holobiont. Although our understanding of the role endosymbiotic microbes play in coral health has advanced, the role surface-associated microbes and their chemical signatures play in coral health is limited. Using minimally invasive water sampling, we show that the corals *Acropora* and *Platygyra* harbor unique bacteria and metabolites at their surface, distinctly different from surrounding seawater. The surface metabolites released by the holobiont create concentration gradients at 0–5 cm away from the coral surface. These molecules are identified as chemo-attractants, antibacterials, and infochemicals, suggesting they may structure coral surface-associated microbes. Further, we detect surface-associated metabolites characteristic of healthy or white syndrome disease infected corals, a finding which may aid in describing effects of diseases.

## Abstract (web summary)

Michael Ochsenkühn et al. look at the microbial and metabolic composition of coral surfaces and the surrounding seawater. They find that the metabolites found on the surface of the coral create a concentration gradient that influences the surrounding microbiome.

## Introduction

Unlike many animals in the oceans, corals lack the ability to change their habitat when faced with unfavorable conditions and must adapt to changes in their environment (e.g., temperature, salinity, and light) in order to survive^1^. In ecosystems like the Great Barrier Reef, and other reefs worldwide, failure to acclimate to such extreme conditions leads to the demise of whole coral communities^2,3^.

Corals and their complex symbiotic communities are often referred to collectively as the holobiont, with a mucus layer as the first line of defense against perturbations from the immediate environment. Coral mucus generally consists of a gel-like matrix of glycoproteins (e.g., mucins), polysaccharides, and lipids^4^, which is constantly regenerated by the animal host^5^. This layer serves numerous functions including sediment cleansing, protection against UV damage and desiccation, growing substrate for bacteria and protection from pathogens and other stressors^4–6^. The mucus layer harbors a diverse microbial community that includes bacteria, viruses, archaea, and some fungi while simultaneously allowing the influx of the symbiotic dinoflagellates, *Symbiodinium*^7–12^.

During stress the chemical composition of the coral mucus changes^13^ concomitant with a shift in the composition of the mucus microbiome^14^. Changes to this microbial community are believed to be due to the invasion of pathogenic microbes and immune deficiency of the coral holobiont^10,12,15–18^. Under normal conditions, the dinoflagellate symbiont produces essential sugars that are used in coral mucus production and regeneration; however, this symbiont is expelled during bleaching events at high temperatures^4,19^. These observations suggest that environmental stress often leads to the disruption of this mucus layer and/or its resident microbiome, which could ultimately be detrimental to the coral host.

A major cause leading to the decline of coral populations is the increase of a wide variety of coral diseases, with diverse morphologies and etiologies, such as white syndrome and black band or yellow band diseases^16–18^. Although the mechanisms of coral infections are still unknown, several microbes have been shown to be implicated in coral diseases^20,21^. For example, cyanobacteria that dominate the microbiome of corals infected with black band disease produce quorum sensing inhibitors that may disrupt communication between members of the indigenous microbiome^22^, leading to a potential disruption of a stable coral microbiome and weakening of the holobiont system. On the other hand, white band disease, a type of white syndrome diseases, though widely investigated to have rapid disease progression^23^ and high infection rates in corals under stress^24,25^, has an unclear cause of infection.

The profile of intracellular metabolites in corals varies depending on environmental factors, such as increases in oceanic temperature and pH as reported in *Pocillopora damicornis*^26^. Corals also show specific shifts in abundance of ubiquitous metabolites^27^ and produce molecules that allow self/non-self-recognition, e.g., specific lipids that lead to a defensive immune response^28^. Coral chemical ecology has been examined with respect to either the microbial communities within coral tissues or in the mucus layer. The best-studied example is dimethylsulfoniopropionate (DMSP), which is widely recognized as one of the early indicators of heat stress and has been shown to function as a chemo-attractant for coral-associated microbes and coral pathogens^29–31^. Corals have also been shown to release amino acid derivatives that may attract bacteria in seawater and structure their microbial community^30,32^. In fact, interactions between the coral and its microbiome and between members of the microbiome, likely mediated by chemical signaling, may be major drivers of adaptation to environmental changes^22,31^. However, the distribution and abundance of nutrients and infochemicals near coral surfaces and their potential importance in structuring the coral microbiome are unknown.

Due to their sedentary nature, we hypothesize that corals and their associated microbiome exude molecules that are first retained in the mucus layer and then slowly diffuse away into the water column. These molecules may help the coral holobiont to attract beneficial microbes or repel harmful ones. Biotic and abiotic stresses to the coral holobiont lead to a change in the coral microbiome that may influence the molecular composition retained in the mucus layer.

Here, we use minimally invasive sampling, meta-barcoding of microbial populations and metabolomics to show that two coral species harbor specific groups of bacteria and classes of molecules on their surfaces that are distinctly different from surrounding seawater. Many of these molecules may play a role in structuring the microbial community around corals. Finally, the minimally invasive sampling reported here can be used to discover candidate biomarkers to help characterize coral disease across species.

## Results

### Microbial gradients surrounding corals

**Fig. 1:**
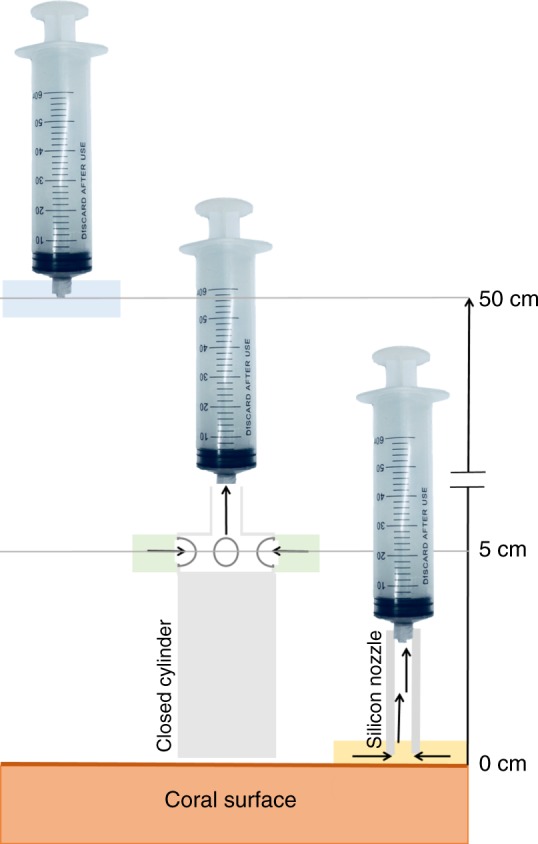
Sketch of the water sampling scheme at 0 cm with a silicon nozzle attached to a syringe, 5 cm with a 5-cm cylinder connected to a syringe with inlets at the base and 50 cm with a regular syringe. Sketch not to scale

Microbial communities associated with corals have been shown to differ from microbes in the surrounding seawater^33^. To confirm this observation in the Arabian Gulf, water samples were collected at various distances from stony coral colonies belonging to *Acropora* (8 colonies) and *Platygyra* (10 colonies) species in the Arabian Gulf over the period of six months (Supplementary Table 1). Colonies included equal numbers of white-syndrome infected^3^ and healthy colonies that were monitored over 2 years. Instead of removing parts of coral colonies and sampling mucus/water by air exposure^4,14^, we collected samples on site using a minimally invasive device from the coral surface as described in the Methods. In brief, coral surface samples (0 cm) were collected with a soft silicon nozzle attached to a syringe, coral vicinity samples (5 cm) were collected with a 5-cm capped cylinder connected to a syringe with inlets at 5 cm above the coral surface and seawater samples (50 cm) were collected with a regular syringe placed ~50 cm away from the coral colony (Fig. 1). We reasoned that 0 cm and 5 cm samples may possess microbial communities different from 50-cm samples.

**Fig. 2:**
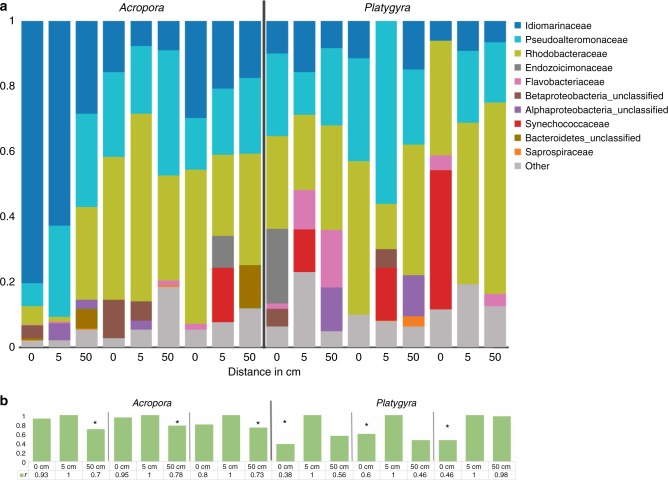
Analysis of the bacterial populations surrounding coral samples. **a** 16S rDNA profiles of bacteria excluding *Pelagibacterales* from the individual distances 0, 5, and 50 cm of *Acropora* and *Platygyra* corals. Three samples from each coral species at each distance were pooled to achieve enough DNA for sequencing. Family level taxonomy was determined with Greengenes and NCBI microbial nucleotide Blast. **b** Bar charts depicting Pearson’s rank correlation (r) between individual distance OTU abundances compared with 5 cm samples; 5 cm shows self-correlation (=1) as a reference. Asterisks indicate statistical significance (Student’s *t*-test, two tailed, equal variance, *Acropora p* = 0.0002, *Platygyra p* = 0.0012) within the species groups for the Pearson’s rank correlations

Using meta-barcoding of the bacterial 16S rDNA gene and sequencing using the Illumina MiSeq platform, we characterized the microbial composition of samples collected in March 2017 at distances of 0, 5, and 50 cm (Supplementary Table 2). For all samples, the recovered reads were dominated by *Pelagibacterales* (~97–99%), one of the most abundant prokaryotes in the marine environment^34^ and a common group of bacteria in coral mucus and surrounding seawater^16,35^. We reasoned that the dominance of *Pelagibacterales* would interfere with any interesting patterns of other bacterial abundance at the sampled distances; thus, we examined the composition of the 10 most abundant bacterial operational taxonomic units (OTUs) after removing *Pelagibacterales* reads. The remaining bacterial OTUs were dominated by *Endozoicomaceae*, a suspected bacterial symbiont of corals, *Flavobacteriaceae, Idiomarinaceae*, and *Rhodobacteraceae*, all of which have been observed in association with corals^36,37^ (Fig. 2a). Across all *Acropora* samples, Pearson’s rank correlation (r) shows a stronger correlation between bacterial diversity and abundance of 0 and 5 cm compared to 5 and 50 cm samples (Fig. 2b). This pattern suggests specific bacterial OTUs concentrate at (0 cm) and near (5 cm) the branching coral surface and eventually intermix with bacterial populations from surrounding seawater. However, in *Platygyra* samples the stronger correlation between 0 and 5 cm vs 5 and 50 cm samples is lost perhaps due to stronger mixing around the more exposed structure of this brain coral species (Fig. 2b). To examine the subtleties of microbial communities’ distribution between both coral species and to assess the differences in chemical composition in such communities of these regions, we collected seawater for mass spectrometry analysis of metabolites from 0, 5, and 50 cm distances as described before.

### Molecular gradients surrounding corals

Samples were collected as described above during August and October 2016 and were filter sterilized and extracted using solid-phase extraction immediately after collection. Mass spectrometry data were acquired using two instruments: an electrospray ionization time-of-flight mass spectrometer coupled to a liquid chromatography system (LC-QToF-MS), which exhibits high sensitivity towards molecules present at lower concentrations and a Fourier-Transform ion cyclotron resonance mass spectrometer (FT-ICR-MS), which provides ultra-high resolving power and allows for increased confidence in putative molecular identification, was performed after a secondary desalting step.

**Fig. 3:**
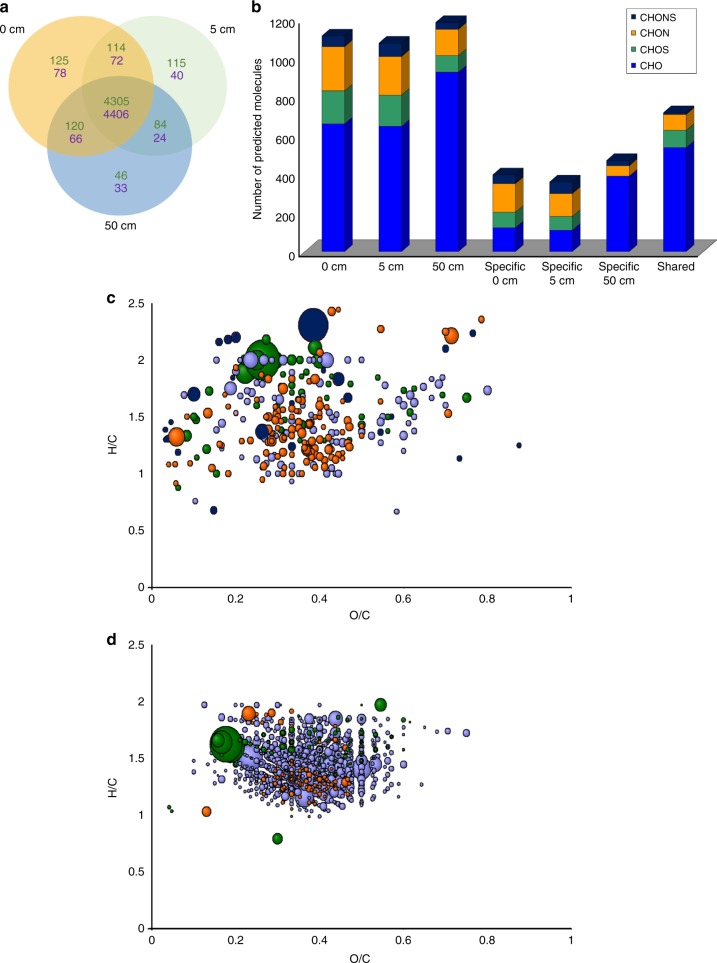
Analysis of molecular features shared among all coral samples and their elemental composition. **a** A schematic Venn diagram of detected molecular features using the LC-QToF-MS at 0, 5, and 50 cm from *Acropora* sp. (green) or *Platygyra* sp. (purple). **b** Bar chart showing the elemental diversity in chemical formulae at the three-distance sample sets of *Acropora* sp. as predicted by their FT-ICR-MS exact masses and isotopic fine-structure patterns. The bar graphs show the total number of compounds and their predicted elemental composition. **c**, **d** Van Krevelen diagrams showing H/C vs O/C ratios of specific compounds unique to 0 and 5 cm only (**c**) or common to 0, 5, and 50 cm (**d**). Size of dots corresponds to compound abundance extracted from measured total ion current. Colors correspond to the elemental composition in panel (**b**)

We detected 4909 and 4719 molecular features from the *Acropora* and *Platygyra* samples using the LC-QToF-MS, respectively, of which 4033 were shared between both species (Fig. 3a). In the *Acropora* and *Platygyra* samples, 88 and 93% of the species-specific features were detected at all three distances, respectively. The remaining features were either unique to one sample or shared in two of three sampled distances (Fig. 3a). Using the FT-ICR-MS, molecules detected in samples from each distance exhibited low variation in total number (~1200 molecules per distance) but differing diversity of elemental composition (Fig. 3b). The elemental composition from the FT-ICR-MS was predicted from molecular formulae calculated from exact masses and isotopic fine-structure patterns (see Methods). Particularly, 50 cm samples showed the highest diversity of dissolved organic carbon molecules (CHO). Samples at 0 and 5 cm showed higher numbers of organic sulfur and nitrogen molecules (CHON and CHOS). Molecules unique to 0 and 5 cm (>300 molecules each) were enriched mostly in CHON species, while the most abundant molecules in these samples belonged to CHOS and CHONS species (Fig. 3c). The van Krevelen diagram of molecules common to all three distances displayed a pattern characterized by a carboxylic-rich alicyclic matter range which is characterized by H/C ratios of ~0.7:1.5 and O/C ratios of ~0.2:0.7 (Fig. 3d), typical of seawater (Supplementary Figure 1)^38,39^.

**Fig. 4:**
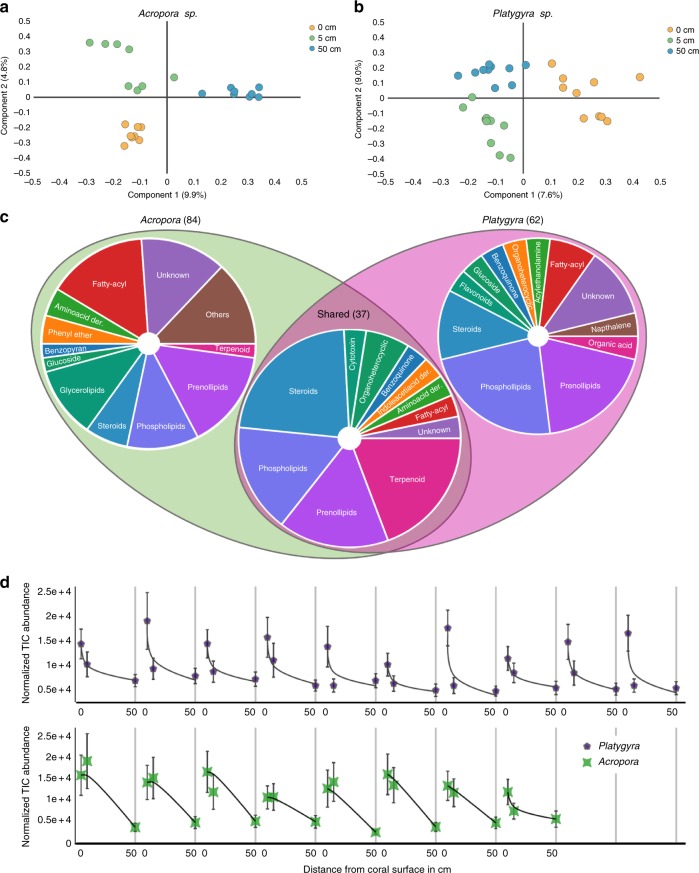
Analysis of metabolite distribution and composition. **a**,**b** Partial least squares discriminant analysis of metabolite samples according to 0, 5, and 50 cm in *Acropora* (**a**) and *Platygyra* (**b**) species. **c** Classification based on putative annotation of all molecular features forming abundance gradients along the vertical sample transect (0 ≥ 5 > 50, with ANOVA *p* ≤ 0.0086 and Welch’s *t*-test *p* ≤ 0.0098) either shared between both species or found only in *Acropora* or *Platygyra* samples. Numbers in parentheses indicate the numbers of molecules in each group. Refer to Supplementary Materials and Supplementary Table 4 for complete putative annotations, *p*-value significance and full mass spectrometry data. **d** Average normalized abundance profiles of the 37 molecular features shared in both *Platygyra* (10 colonies) and *Acropora* (8 colonies) species across all distances. For each profile, plotted points represent the normalized abundance at 0, 5, and 50-cm distances (left to right); lines represent fitting of the abundances to an exponential decay function. The vertical grey  lines separate the profile of each colony. Error bars represent single standard deviations of 30 normalized intensities of the individual compounds as annotated in Supplementary Table 4

Partial least squares discriminant analysis, a supervised method for principal component detection^40^, was performed on all features with sample groupings 0, 5, and 50 cm. This analysis showed a distinct clustering of biological replicates at each sampling distance with the first three components accounting for 21% of the variance in *Acropora* and 25% in *Platygyra* samples (Fig. 4a,b, Supplementary Figure 2). Particularly, 0 cm samples from both species clustered together and were strongly separated from both 5 and 50 cm samples, a pattern that was especially pronounced for *Acropora* samples (Fig. 4a). A similar pattern of a more distinct composition of 0 and 5 cm relative to 50 cm around *Acropora* relative to *Platygyra* is also observed in the distribution of bacterial phylotypes (Fig. 2b). These observations suggest that coral surfaces possess unique molecular and microbial signatures distinctly different from seawater.

Many of the detected molecular features are prominent infochemicals in coral reefs. These include the coral gametogenesis hormone estradiol-17*β*^41^, several defense molecules including the diterpenes flexibilide and dihydroflexibilide produced by reef dominant soft corals that are toxic to fertilized *Acropora* eggs and sperm^42^, several bacterial quorum sensing molecules (e.g., coumaroyl-homoserine lactone), several quorum sensing inhibitors (e.g., bromofuranone)^43,44^, and antibacterial compounds (e.g., malabaricone C). In addition, we detected many central metabolites including carbohydrates (e.g., arabinonic acid) and amino acids (e.g., valine, lysine, leucine, tyrosine) (Supplementary Table 3). These nutrients, infochemicals, and toxins may potentially play specific roles in structuring the holobiont community or influencing coral health.

In the *Acropora* and *Platygyra* samples, 306 and 286 molecules, respectively, showed statistically significant differences in relative abundances between 0, 5, and 50 cm across all biological replicates (ANOVA *p* ≤ 0.0086). Among these molecules, 84 and 65 molecules showed higher abundances at 0 cm relative to 5 and 50 cm in the *Acropora* and *Platygyra* samples, respectively (0 ≥ 5 > 50, with ANOVA *p* ≤ 0.0086 and Welch’s *t*-test *p* ≤ 0.0098, Bonferroni tested). Classification of these molecules according to their putative annotations shows a marked difference between the abundance of specific classes of molecules between both species of corals. Phospholipids, prenolipids, and steroids dominated the *Platygyra* 0 cm samples while prenolipids, phospholipids, glycerolipids fatty-acyl molecules dominated the *Acropora* 0 cm samples (Fig. 4c). Thirty-seven of these molecules were shared in both species (Fig. 4c) and had either higher relative abundances at 0 cm followed by a gradual decrease to 5 cm and then 50 cm or showed higher abundance at both 0 and 5 cm followed by a sharp decrease in abundance at 50 cm, resembling a diffusion profile (Fig. 4d). Therefore, we hypothesized that these 37 molecules are produced by the coral holobiont and gradually diffuse from the surrounding mucus layer. Using high-resolution mass-to-charge ratios (*m/z*), exact mass isotope patterns obtained from the FT-ICR-MS together with tandem mass spectrometry (MS^2^) and additional support by in silico fragment prediction, 30 of the 37 molecular features released by the coral holobiont were putatively annotated (Supplementary Table 4). Most of these molecules belong to the phospholipid, steroids, steroid-glucoside, prenolipids, and terpenoid chemical classes (Fig. 4c), which are abundant in corals^45–47^. Interestingly, triterpenes and other terpenes from several sponges and macroalgae have been shown to disrupt coral symbiosis with dinoflagellates by increasing respiration rates of adjacent corals and interfering with the photosynthetic capabilities of dinoflagellates^48,49^. Additionally, several byproducts of the coral gametogenesis hormone estradiol-17*β*, 2-hydroxyestradiol, and methoxy-estradiol, were detected (Supplementary Table 4) in increased amounts close to the coral surface.

### Coral disease indicators

**Fig. 5:**
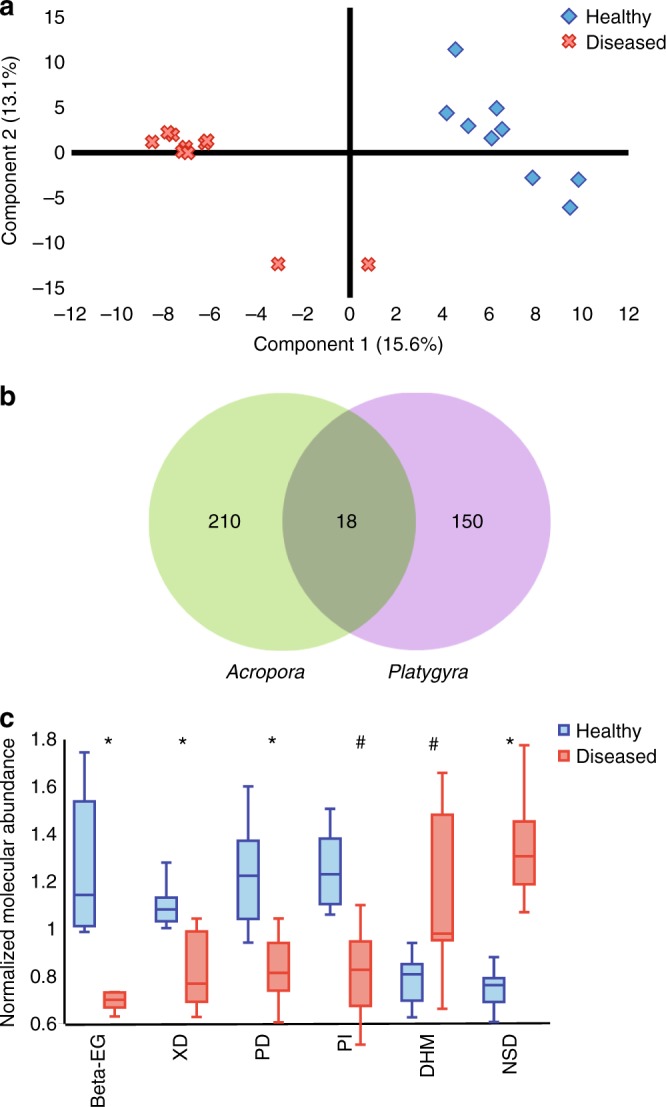
Defining coral disease indicators. **a** Partial least squares discriminant analysis of CS molecular features from healthy and diseased corals. **b** Schematic Venn diagram showing unique and shared molecular features from 0-cm samples of *Acropora* and *Platygyra* colonies. **c** Box and whisker plot showing candidate indicators with similar normalized abundance trends common to both coral species. The mid line depicts the median, box edges the lower and upper quartiles and whiskers min/max observed values. Statistically significant trends (Welch’s *t*-test) between healthy and diseased normalized abundances across colonies are marked with asterisk (*p* ≤ 0.00059) or hash (*p* < 0.0082). Error bars represent minimum and maximum values of the sample group. Beta-EG = 17-*β*-estradiol-3-glucuronide, XD = xanthine derivative, PD = pyridine derivative, PI = phosphatidyl-inositol, DHM = dihomomethionine, NSD = Norspermine derivative

White band disease is a common coral disease observed at high temperatures throughout the Pacific Ocean and the Arabian Gulf and is manifested with tissue loss at the lesion site that exposes the coral skeleton^21^ (Supplementary Figure 3). White band disease causes rapid tissue loss during the hot summer months in the Arabian Gulf in both *Acropora* and *Platygyra*^3^, spreading a few millimeters daily^23^. Although infected corals in this study exhibited tissue loss characteristic of this disease, we have not confirmed this and thus refer to these colonies as suffering from a white syndrome disease. Only 0 cm samples were compared across healthy and diseased colonies with the assumption that metabolites that characterize diseased and healthy colonies would be concentrated near the surface. Partial least squares discriminant analysis showed a distinct separation of healthy and diseased samples (Fig. 5a), suggesting that there is indeed a chemical signature unique to surfaces of diseased and healthy corals. The number of features with significant (ANOVA, *p* ≤ 0.038) differential presence between healthy and diseased colonies of *Acropora* and *Playtgyra* were 238 and 168, respectively (Fig. 5b). Of 18 molecules that were common to both species (Fig. 5b), six molecules exhibited statistically significant differential abundances between healthy and diseased samples (Fig. 5c). These molecules were either more abundant in all diseased coral colonies examined or more abundant in all healthy colonies from both species. Molecules that were more abundant on healthy coral surfaces were 17-*β*-estradiol-glucuronide, xanthine- and pyrimidine-derivatives, and phosphatidyl-inositol suggesting that they may serve an important role in either disease resistance or regulating bacterial colonization of coral surfaces (Fig. 5c, Table 1). Dihomomethionine and a norspermine derivative were more abundant in diseased corals, possibly playing a defense mechanism role (Fig. 5c).

**Table 1:** Putative annotations for candidate white syndrome disease indicators

Putative compounds	Chemical formula	Mono-isotopic mass	PubChem	LC/MS (+)	FT-ICR (−)		
				Adduct	Δ ppm	adduct	Δ ppm
17-*β*-Estradiol-3-glucuronide	C_24_H_32_O_8_	448.2097	HMDB0010317	[M2 + NH_4_]	9	[M-H]	0.1
Xanthine derivative	C_17_H_20_N_4_O_2_	312.1586	128784	[M + H]	11	[M-H]	2.6
Pyridine derivative	C_20_H_33_N_9_O_2_	431.2754	90211990	[M + H]	6.7	[M-H]	2.5
Phosphatidyl-inositol	C_47_H_88_O_16_P_2_	970.5547	CHEBI:17526	[M + H + NH_4_]	10	NA	NA
Dihomomethionine	C_7_H_15_NO_4_S	209.0722	90657203	[M + H]	4	[M-H]	0.4
Norspermine	C_14_H_34_N_6_O	302.2794	20681656	[M + H]	4	[M-H]	0.2

## Discussion

Our results show that two coral species that possess starkly different phylogenies and skeletal structures are surprisingly surrounded by a sphere of characteristic bacteria and shared metabolites. Our results show that the microbial diversity in water samples surrounding the coral colonies varies at different distances (Fig. 2). Predictably, the most dominant microbes in all samples were *Pelagibacterales* (SAR 11), the most abundant bacterium in the marine environment^50^. A core 0/5-cm microbiome was not apparent for either *Acropora* or *Platygyra* species (Fig. 2). Interestingly, microbial populations seemed to be retained more strongly on *Acropora* colonies than on *Platygyra* colonies (Fig. 2b), which could be due to different fluid dynamics caused by the branching and brain type coral structures.

The chemical composition of the coral surface (0–5 cm) is characterized by an increasing chemical diversity and abundance of heteroatom (N, O, S)-containing organic molecules, which suggests that these dissolved organic sulfur and nitrogen molecules are biologically-derived from the coral holobiont (Fig. 3). The presence of fewer heteroatom-containing molecules in seawater relative to 0 and 5 cm may be due to the consumption of these molecules by microbes in seawater and/or diffusion of these molecules away from the coral holobiont, which leads to dilution below our detection limit. In contrast, 50-cm samples displayed more CHO species that likely represent dissolved organic carbon, like complex carboxylic-rich alicyclic molecules (Fig. 3, Supplementary Figure 1).

Although the uniformity and size of this sphere of molecules surrounding the coral holobiont are uncertain and likely vary depending on shear in seawater, our analysis consistently detect diffusion-like patterns from the surface of corals outwards up to 5 cm away, where concentrations of molecules are still significantly above background levels (50 cm) (Fig. 4). Movement of epidermal cilia covering coral surfaces has been shown to enhance transfer of gases and molecules in the microenvironment of corals^13,51^, which may partially account for the observed bacterial and molecular patterns observed near coral surfaces. However, cilia movement is estimated to increase mass transfer ~1–2 cm away from coral surfaces, which does not fully account for the observation of molecular diffusion up to 5 cm away from coral surfaces. Interestingly, the outermost layer of corals tissue consists of a thin mucus layer that hosts a unique microbial community^14,44^, an observation that is corroborated by our bacterial community profiles at 0 and 5 cm (Fig. 2). This layer likely retains chemical compounds released from either host or microbial community and allows for slow but constant diffusion into the environment. This diffusion can then produce a concentration gradient close to the holobiont surface, which is measured in this work.

The existence of molecular gradients around coral holobionts is analogous to diffusive boundary layers around plants roots (i.e., the rhizosphere)^52^ and phytoplankton cells (i.e., the phycosphere)^53^. Like these regions, the presence and composition of a holosphere around corals may be essential for the coral host and potentially indicative of its health state. For example, metabolite excretion by the coral and retention within this region may help recruit symbionts and beneficial microbes to the coral host (e.g., amino acid derivatives)^30^. Likewise, signaling molecules and antimicrobial compounds must be retained in an equilibrium at sufficient concentrations to allow beneficial microbes to properly colonize the coral surface or to fend off harmful microbes. The patterns of bacterial (Fig. 2) and molecular abundances (Fig. 4) and identity of the molecules detected in this region support this depiction (e.g., central metabolites, hormones, antimicrobial molecules, and quorum sensing molecules) (Table 1, Supplementary Tables 3, 4). Many of these molecules produced by the coral holobiont are hypothesized to play an important role in structuring the coral surface and mucus microbial composition.

The molecular diffusion profiles observed between the 30 shared molecules found in *Acropora* and *Platygyra* samples exhibit different behavior as a function of distance from the coral surface. For example, *Acropora* diffusion profiles show an average drop of 7% (±5.3%) in molecular abundance from the surface to 5 cm whereas the same metabolites drop by an average of 47% (±4.2%) for *Platygyra* (Fig. 4d). This large difference may be attributed to differentially altered fluid dynamics due to structural and morphological differences between both corals. *Acropora* forms branched structures with large surface areas that may lead to better retention of molecules and bacteria, whereas *Platygyra* forms uniform, brain-like structures that are prone to seawater turbulence and thus may be relatively inefficient at retaining molecules or bacteria (Supplementary Figure 2). These structural attributes of different coral species may be indicative of divergence in species-specific adaptation. Further work is needed to examine how these morphological differences influence the fluid dynamics surrounding corals and how this, in turn, affects microbial and chemical exchanges between the coral holobiont and surrounding waters.

Coral diseases are widespread owing to anthropogenic interference and rising temperatures. Understanding the mechanisms of these diseases is essential to support coral survival through coastal management efforts^54,55^. The detection of candidate disease indicators using minimally invasive methods represents a valuable tool to detect pathogens and molecules responsible for this disease, as well as others and maybe used to aid etiology studies. We hypothesized that specific molecules in 0 cm samples can serve as candidate biomarkers or disease indicators by comparing samples of *Acropora* and *Platygyra* colonies that were either healthy or exhibited a white syndrome disease. In this study, six potential indicator metabolites for *Acropora* and *Platygyra* suffering from a white syndrome disease have been detected (Table 1). Although the role these molecules play in disease, if any, is not clear, their consistent presence suggests an essential role in either infection or response to the disease by the holobiont. Diseased colonies showed increased levels of dihomomethionine and a norspermine derivative. Dihomomethionine is a methionine derivative and precursor for several metabolites in the glucosinolate biosynthesis pathway, which comprises stress-signaling metabolites in green plants involved in heat shock protein production and host-pathogen recognition^56,57^. The elevated levels of dihomomethionine found in diseased corals may indicate a stress signal in response to an active infection (Fig. 5c). Norspermine, a polyamine, is also a methionine derivative that shows elevated levels in diseased colonies. Norspermine and other polyamines play important roles in abiotic stress responses and have been shown to disrupt biofilm formation in Gram-positive bacteria, a mechanism that may explain its relatively high abundance in diseased corals (Fig. 5c)^58,59^. The significantly low abundance of 17-*β*-estradiol-glucuronide, xanthine- and pyrimidine-derivatives, and phosphatidyl-inositol around diseased colonies indicates a change in the metabolism of the coral that is struggling to cope with either infection or other environmental stresses. In addition, the higher abundance of 17-*β*-estradiol-glucuronide, a byproduct of the coral sex hormone 17*-β*-estradiol, in healthy corals indicates that sex hormones are not only important in coral propagation during spawning events^60^ but that downregulation of constitutive hormone pathways may be indicative of coral stress, as reported for UV exposed coral larvae^61^. This observation is especially important since other products of the estradiol pathway were also detected (Table 1, Supplementary Tables 3, 4).

Our findings support the presence of a small molecular environment surrounding corals, which could be part of the coral signaling and defense pathways. Since coral surfaces represent the first line of defense against disease, healthy corals may maintain beneficial or healthy microbial communities by secreting a combination of antibacterial and chemo-attracting molecules to keep these microbial communities in check. At the onset of disease, the metabolism of the coral holobiont shifts from maintaining a healthy microbial community at the surface to likely fighting the disease, which may include preventing biofilm-forming pathogens from colonizing the colony surface among other mechanisms.

Further work is needed to characterize the temporal and spatial variations in the chemical and microbial composition of the holosphere to identify the sources of molecules that play an important role in this region. The present in situ work shows the possibility of defining specific candidate disease indicators for a coral disease in two coral species using minimal invasive sampling on site; however, future work needs to include a wider range of species from diverse geographic locations to verify potential global chemical markers. Such studies will facilitate our ability to potentially predict coral disease before its occurrence and perhaps develop mechanisms to combat such diseases especially as corals face global extinction.

## Materials and methods

### Sampling

Samples were collected around Saadiyat Reef, Abu Dhabi, UAE, (N 24°35′54.2″, E 54°25′12.9″) by SCUBA from either *Acropora* sp. or *Platygyra* sp. corals at a depth of 6–7 m. Sampling for DNA meta-barcoding was conducted in March 2017 while sampling for metabolome samples was conducted in August and October 2016. Environmental parameters (GPS coordinates, temperature, salinity, pH, dissolved oxygen, turbidity, and redox potential) were measured with a YSI Multimeter (Xylem, US) at each sampling site (Supplementary Table 1). Seawater samples were collected from three different distances: 0 cm (coral surface), 5 cm, and 50 cm (seawater control) away from the coral surface. A sketch of the sampling can be found in Fig. 1. Samples were collected with 50 mL syringes at 10–20 random spots, only from non-infected visibly viable tissue containing parts on the same coral colony, withdrawing 2.5–5 mL from each spot. Eight and ten replicate samples from each distance, 0 cm, 5 cm, and 50 cm from the coral surface, were taken from 8 and 10 different tagged and monitored *Acropora* and *Platygyra* colonies, respectively. Both species contained 50% visibly healthy and 50% white-syndrome diseased colonies. Samples were filter sterilized immediately onboard the boat using sterile 25 mm 0.2-µm nylon filters (Phenomenex, US) and kept on ice in the dark until compound recovery on the same day.

### 16S rDNA sequencing

For the collection of microbes, three 50 mL seawater samples were combined from each distance prior to filtering on 47 mm 0.2-µm nylon filters (Whatman, UK) in SwinLok filter holders (Sigma, Germany). Filters were stored in 100% Ethanol on ice until DNA extraction in the laboratory on the same day. DNA was extracted using the Qiagen All-prep-Kit (Qiagen, US) according to the manufacturer’s instructions. Recovered DNA was amplified with forward primer V4_515F_New (5′-GTGYCAGCMGCCGCGGTAA) and reverse primer V4_806R_New (5′-GGACTACNVGGGTWTCTAAT) by Fluidigm (Fluidigm Co., CA, USA) and sequenced using MiSeq 2 × 300 (Illumina, CA, USA) paired-end library at the Carver Biotechnology Center (University of Illinois-Urbana Champagne, USA).

Sequences were analyzed using the Mothur platform^62^. After contig alignment and trimming, identical sequences were merged using the ‘unique.seqs’ command to save computation time, and the command ‘count.seqs’ was used to keep a count of the number of sequences over samples represented by the remaining representative sequence. Rare sequence reads were removed (*n* < 2) and the remaining sequences were aligned against the SILVA database (release 128)^63^. Chimeric sequences were removed using UCHIME as implemented in MOTHUR^64^. Chloroplasts, mitochondria, eukaryotes, and unknown reads were also removed. Sequences were classified against Greengenes using bootstrapping of 60, and the sample compositions were compared on the family level^65^. For further analyses, a 97% similarity cut-off level was chosen to obtain OTUs. After trimming, singleton and chimera removal, and chloroplast filtering, the sequencing resulted in 2.15 × 10^6^ reads from 18 samples. Samples contained a minimum of 89,934 reads and a maximum of 140,127 reads (Supplementary Table 2).

### Compound recovery

All glassware used for sample processing were baked at 420 °C for 24 h. All samples were directly loaded onto HLB solid-phase extraction columns (60 mg, Waters, US) that were conditioned according to the manufacturer’s instructions. Samples were loaded onto the columns using a multi-channel peristaltic pump (Masterflex, US) at 1 mL per min. Subsequently, columns were washed with 20 column volumes of ultra-pure water (MilliQ-H_2_O) and stored at −80 °C until elution. All collected samples were eluted on the same day with 2 mL of 100% methanol (LC-MS grade, BDH Scientific, Dubai, UAE) into glass tubes. The methanol was removed by evaporation using a Savant Speedvac (ThermoFisher, CA) at room temperature. The dry metabolites were re-dissolved in methanol with 0.1% formic acid.

### UHPLC-Q-ToF-MS metabolic profiling

Metabolites were analyzed using an Agilent 1290 HPLC system coupled to an Agilent Technologies 6540 Accurate Mass Q-ToF-LC/MS (Agilent, US). Metabolites were separated using a reversed-phase separation method. In reverse-phase mode, medium-polarity and non-polar metabolites were separated using an Eclipse Plus C_18_ column (50 mm × 2.1 mm ID) (Agilent, US). Chromatographic separation consisted of MilliQ-H_2_O + 0.1% formic acid (buffer A), 30 mM ammonium formate in MilliQ-H_2_O (buffer B) and Acetonitrile + 0.1% formic acid (Buffer C). The gradient started with 90% A, 5% B, and 5% C, with an initial gradient of 2 min to 65% A, 5% B, and 30% C. The secondary gradient to 100% C after 10 min and held for 2 min. The column was allowed to equilibrate for 5 min in the starting conditions. Detection was carried out in the positive ionization mode with the following parameters: ESI settings: dry gas temperature = 350 °C, dry gas flow = 8.0 l/min, nebulizer pressure = 35 psi, VCap = 2100 V, end plate; MS-ToF setting: fragmentor = 175 V, skimmer = 65 V, and Oct 1 RF Vpp = 750 V, (collision energy for full scan MS = 0, targeted MS^2^ = 10, 30, 50 eV); acquisition setting: mass range = 60–1400 *m/z*, rate 1 spectra/s, 1000 ms/spectrum.

### FT-ICR-MS metabolic profiling

FT-ICR-MS allows the measurement of high-resolution accurate mass and isotopic fine structure for direct determination of the chemical formula of a compound. FT-ICR direct infusion measurements are prone to salt contamination, therefore a second purification step with PPL-bond elute SPE (Agilent Technologies, US) was applied. High-resolution mass spectra were acquired on a Bruker solariX Ion Cyclotron Resonance Fourier Transform Mass Spectrometer (FT-ICR-MS) (Bruker Daltonics GmbH, Germany) equipped with a 12T superconducting magnet (Magnex Scientific Inc., UK) and an APOLO II ESI source (Bruker Daltonics GmbH, Germany) operated in the negative ionization mode. The negative ion mode mass spectra allow for a smaller number of adducts, as well as higher resolution compared to positive ionization in the FT-ICR-MS. Spectra were internally calibrated using NOM (natural organic matter) internal reference and were acquired with a time domain of 4 mega words over a mass range of *m/z* 100 to 1000, with an optimal mass range from 200–600 *m/z*. Three-hundred scans were acquired for each sample. The FT-ICR mass spectra were exported to peak lists with a cut-off signal-to-noise ratio (S/N) of 4. Peak alignment was performed with maximum error thresholds of 0.01 ppm and filtered for masses occurring at a minimum of 50% of the samples. Chemical formula calculation was performed with an error threshold of 0.5 ppm from the exact mass for the chemical formula and isotopic fine structure. Chemical formulae were only generated if all theoretical isotope peaks (100%) were found in spectra.

### Mass spectrometry data processing and statistical analysis

Calibration, alignment, and peak picking of individual LC-MS runs were performed using the Genedata Expressionist for MS 10.0 software (Genedata AG, Basel, Switzerland). Individual data pre-processing steps, peak and retention-time alignment was performed in Genedata Refiner. Background noise was removed by applying an intensity counts cut-off of 500. Peak picking and integration were combined with ^13^C cluster detection for molecular feature verification. All statistical analyses were performed with Genedata Expressionist for MS 10.0 (Genedata, Basel, Switzerland). The acquired LC-MS data was scaled across all samples^66^. Principal least squares discriminant analysis was used to analyze all samples in order to reduce the complexity of the datasets, which is a preferable analysis to principal component analysis in the case of multiple related sample groups^40^. ANOVA and Welch *t*-tests were employed to detect molecular features with significant abundances below *p* < 0.01. Bonferroni and Post-hoc tests were employed to decrease false discovery rates.

Molecular feature identification was based on LC-MS accurate mass and targeted MS^2^ fragmentation analysis of annotated features. Confirmation of molecular formulae and identities was performed with Sirius 3.2 in silico fragmentation software Suit (Uni Jena, Germany)^67^.

## Electronic supplementary material

Supplementary Material

Description of additional supplementary items

Supplementary Data 1

Supplementary Data 2

## Data Availability

The mass spectral datasets generated in this manuscript are available in the MassIVE database under accession No. MSV000082930. The 16S rDNA reads generated here are available in GenBank’s short read archive under accession No. SRP154822.
