# Supplementary material for: Coral metabolite gradients affect microbial community structures and act as a disease cue
Source: Commun Biol. 2018 Nov 5;1:184. doi: 10.1038/s42003-018-0189-1 (PMC6218554; doi:10.1038/s42003-018-0189-1)
Supplement: Supplementary file 1 — Supplementary Material [file 42003_2018_189_MOESM1_ESM.docx]

**Supplementary Information for**

**The Scent of Corals – Coral metabolite gradients affect microbial community structures and act as a disease cue**

**
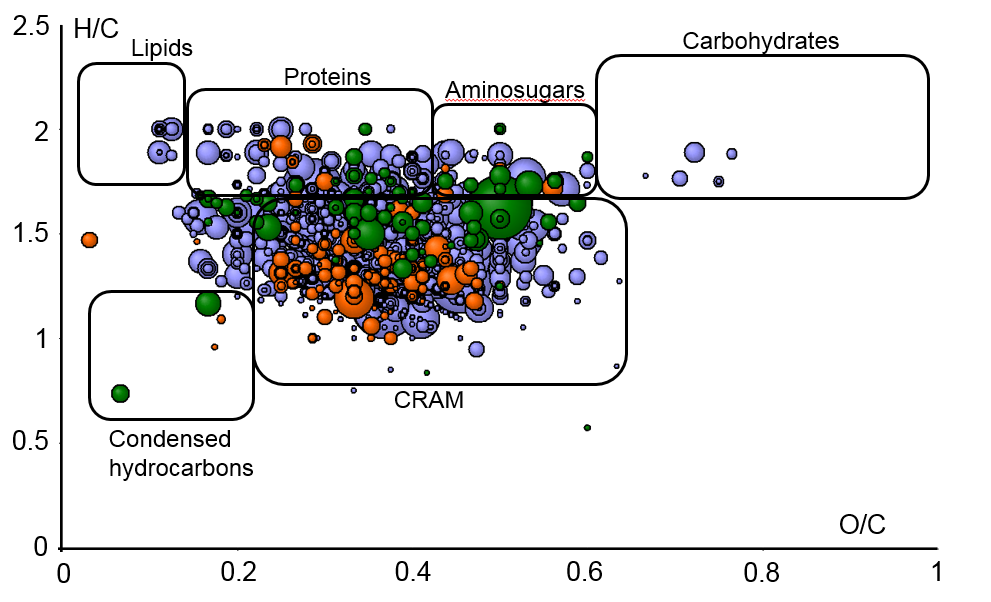
**

**Supplementary Figure 1.** Van Krevelen diagram showing H/C vs O/C ratios of compounds found in seawater (50 cm samples). Boxes describe H/C and O/C ratio ranges characteristic of lipids, proteins, aminosugars, carbohydrates, condensed hydrocarbons and carboxylic-rich alicyclic molecule–like (CRAM) predicted chemical formulae. Size of dots corresponds to compound abundance extracted from measured total ion current. Colors correspond to the elemental composition in panel Fig 3b.

A

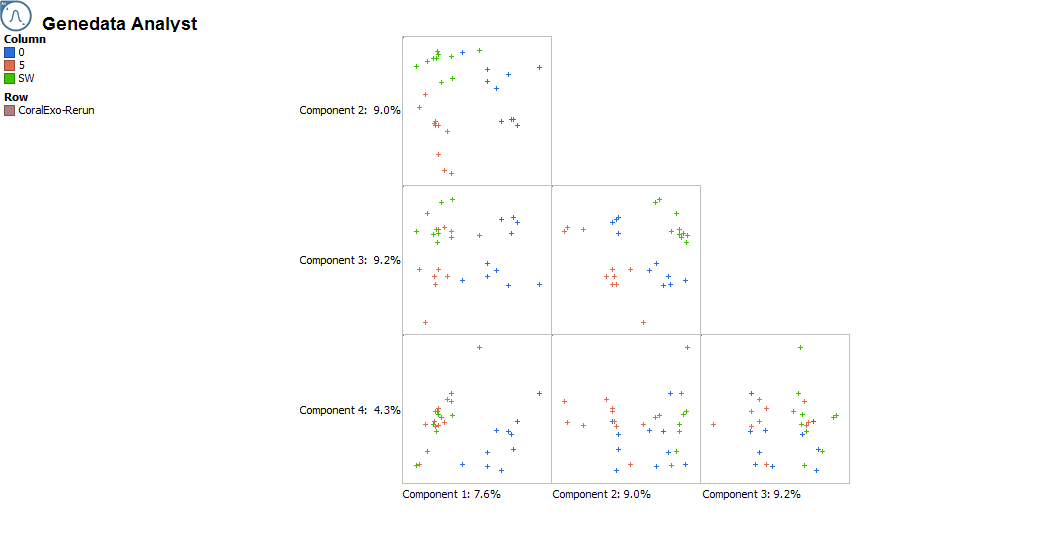
B

**Supplementary Figure 2.** Partial least squares discriminant analysis of the three first components of the analyzing differences between the grouping 0 cm (yellow), 5 cm (green) and 50 cm (SW, blue) in *Acropora sp.* (A) and *Playgyra sp*. (B) samples.

**H**

**Supplementary Figure 3.** Examples of *Acropora sp.* and *Platygyra* sp. corals colonies infected with a white syndrome disease. *Acropora* (A) and *Platygyra* (B) colonies infected with a white syndrome disease (I) and healthy colonies (H). Photos are copyrighted and kindly provided by Dr. David Abrego and Dr. Emily Howells.

**H**

**I**


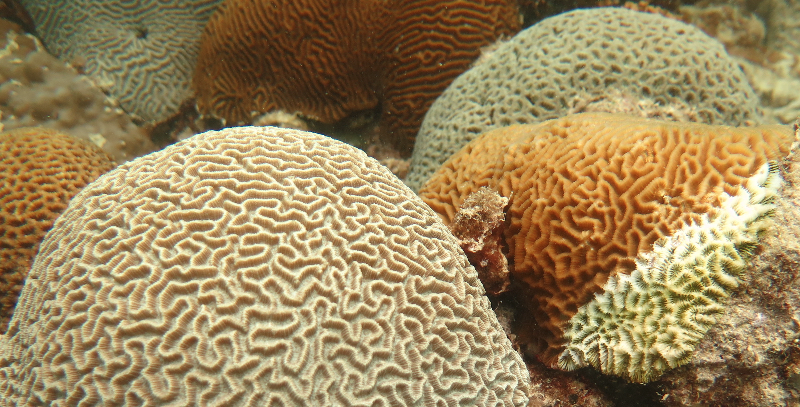


**H**

**I**

A B


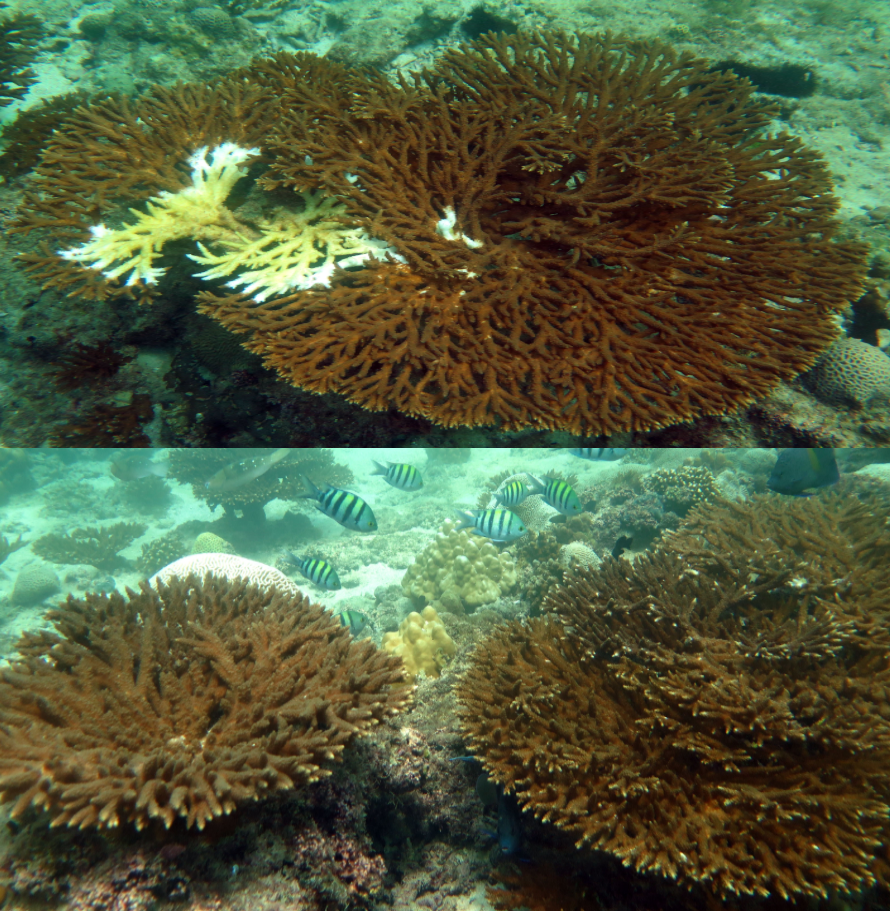


**Supplementary Table 1.** Environmental parameters for water samples. * denotes dissolved oxygen, ^¶^ denotes specific conductance in milli-Siemens per cm and ^†^ denotes total dissolved solids.

| Sample  Type | Date | Latitude | Longitude | Depth (m) | Temperature (°C) | pH | %DO^*^ | Salinity (‰) | SPC^¶^ (mS/cm) | TDS^†^ (g/L) |
| --- | --- | --- | --- | --- | --- | --- | --- | --- | --- | --- |
| Metabolite  *Acropora* | 08/29/16 | 24° 35' 53.5" N | 54° 25' 13.1" E | 6.1 | 34 | 7.9 | 95.8 | 42.2 | 63 | 41 |
| Metabolite  *Platygyra* | 10/27/16 | 24° 35' 55.0'' N | 54° 25' 11.3'' E | 6.8 | 29.9 | 8.0 | 99.2 | 43.1 | 64 | 41.6 |
| DNA  Both sp. | 03/29/17 | 24° 35' 53.5" N | 54° 25' 13.1" E | 6.3 | 24.8 | 8.2 | 98.7 | 42.6 | na | 41 |

**Supplementary Table 2.** Overview of Illumina MiSeq reads counts recovered from 3x 50 mL pooled samples at different distances from March 2017 sampling.

| Species | Sample Name | Sample Distance Label | Total Reads |
| --- | --- | --- | --- |
| *Acropora sp.* | A1 | CS | 158,213 |
|  |  | CV | 192,670 |
|  |  | SW | 177,853 |
|  | A2 | CS | 167,785 |
|  |  | CV | 306,132 |
|  |  | SW | 133,910 |
|  | A3 | CS | 158,512 |
|  |  | CV | 157,125 |
|  |  | SW | 171,932 |
| *Platygyra sp.* | P1 | CS | 114,142 |
|  |  | CV | 108,087 |
|  |  | SW | 148,584 |
|  | P2 | CS | 167,651 |
|  |  | CV | 223,772 |
|  |  | SW | 300,069 |
|  | P3 | CS | 126,532 |
|  |  | CV | 216,973 |
|  |  | SW | 139,197 |

**Supplementary Table 3.** List of putative annotations of molecules of interest detected in metabolome samples along with their ecological relevance.

| Molecule | Bioactivity | Chemical formula | Monoisotopic Mass (Da) | LC/MS (+) | FT-MS (-) |
| --- | --- | --- | --- | --- | --- |
|  |  |  |  | Δppm | Δppm |
| Hexanoyl-L-HL | QS | C_10_H_17_NO_3_ | 199.1208434 | NA | 0.1 |
| N-(3-hydroxydecanoyl)-L-HSL | QS | C_14_H_25_NO_4_ | 271.1783583 | 5 | 0.22 |
| Malabaricone C | Anti-QS | C_21_H_26_O_5_ | 358.1780239 | 4 | 0.04 |
| Catechin | Anti-QS | C_15_H_14_O_6_ | 290.0790382 | NA | 1.36 |
| Tropodithietic acid | ABX | C_8_H_4_O_3_S_2_ | 211.9601854 | NA | 0.5 |
| N-Acetylgalactosamine | Signaling | C_8_H_15_NO_6_ | 221.0899372 | NA | 0.09 |
| Lumichrome | Anti-QS | C_12_H_10_N_4_O_2_ | 242.0803756 | NA | 0.27 |
| Riboflavin | Anti-QS | C_17_H_20_N_4_O_6_ | 376.1382844 | NA | 1.39 |
| Autoinducer-2 (AI-2) | QS | C_5_H_10_BO_7_ | 193.0519581 | NA | 1.36 |
| (S)-4,5-dihydroxy-2,3-pentanedione | AI-2 precursor | C_5_H_8_O_4_ | 132.0422487 | 9 | NA |
| Cinnamoyl-HSL |  | C_13_H_13_NO_3_ | 231.0895433 | 10 | 0.34 |
| Bromofuranone | Anti-QS | C_4_H_3_BrO_2_ | 161.9316422 | NA | 0.16 |
| p-coumaroyl-HSL |  | C_13_H_13_NO_4_ | 247.0844579 | NA | 0.3 |
| Cyclo-L-Leu-L-Val |  | C_11_H_20_N_2_O_2_ | 212.1524779 | 10 | 2.1 |
| Cyclo-L-Phe-L-Pro |  | C_14_H_16_N_2_O_2_ | 244.1211778 | 9 | 2.3 |
| Xf DSF |  | C_15_H_30_O_2_ | 242.2245802 | NA | 0.21 |
| Estradiol | Defense | C_18_H_24_O_2_ | 272.17763 | 9 | 0.08 |
| Flexibilide | Defense | C_20_H_30_O_4_ | 332.14409 | 6 | 0.05 |
| Dihyrdoflexibilide | Defense | C_20_H_32_O_4_ | 336.23006 | 7 | 0.05 |
| Sarcophine | Defense | C_30_H_25_O_3_ | 433.18037 | 4 | 3.16 |
| **Standard Metabolites** |  |  |  |  |  |
| Tyrosine |  | C_9_H_10_NO_3_ | 181.073893 | 3 | 0.15 |
| Valine |  | C_5_H_11_NO_2_ | 117.078979 | 2 | NA |
| Leucine |  | C_6_H_13_NO_2_ | 131.094629 | 4 | NA |
| Lysine |  | C_6_H_14_N_2_O_2_ | 146.105528 | 6 | NA |
| N-Methyl-Proline |  | C_6_H_11_NO_2_ | 129.078979 | 3 | NA |
| S-Methyl-methionine |  | C_6_H_14_NO_2_S | 164.074525 | 4 | NA |
| Histidinol |  | C_6_H_11_N_3_O | 141.090212 | 3 | NA |
| Valyl glycine |  | C_7_H_14_N_2_O_3_ | 174.100442 | 7 | NA |
| Alanyl alanine |  | C_6_H_12_N_2_O_3_ | 160.084792 | 6 | NA |
| Serinyl Valine |  | C_8_H_16_N_2_O_4_ | 204.111007 | 4 | NA |
| Arabinonic acid |  | C_5_H_10_O_6_ | 166.047738 | 8 | 2.57 |
| Adenine |  | C_5_H_5_N_5_ | 145.013698 | 1 | NA |
| Cinnaminic acid |  | C_9_H_8_O_2_ | 148.052429 | 9 | NA |

**Supplementary Table 4.** Putative annotations of molecular features forming a concentration gradient in *Acropora sp.* and *Platygyra sp*. samples with a statistically significant change from 0 and 5 cm to 50 cm (ANOVA and Welch t-test p<0.01). The compounds were either shared in both species (A), or specific to *Acropora* (B) or *Platygyra* (C) samples.

1. Shared Molecular features

| LC/MS  (m/z) | Formula | Adduct | LC/MS (Δppm) | Adduct | FT-ICR (Δppm) | Chemical group | Potential Compounds |
| --- | --- | --- | --- | --- | --- | --- | --- |
| 206.0815 | C_11_H_11_NO_3_ | M+H | 2 | M-H | 0.03 | IAA derivative | 5-Methoxy-Indoleacetate |
| 253.1523 | C_15_H_24_O_3_ | M+H | 0 | M-H | 0.05 | Terpenoid | Elongatol |
| 255.1588 | C_14_H_22_O_4_ | M+H | 5 | M-H | 0.15 | Terpenoid | Laevinol F |
| 303.1938 | C_19_H_26_O_3_ | M+H | 6 | M-H | 0.08 | Steroid | Methoxy-Estradiol |
| 304.2243 | C_7_H_13_NO_2_ | M+H | 4 | NA | NA | AA derivative | Proline betaine |
| 352.2567 | C_37_H_68_NO_8_P | M+H+NH4 | 6 | NA | NA | Terpenoid | Phytocassan B |
| 370.7313 | C_39_H_62_O_12_ | M+H+NH4 | 4 | NA | NA | Steroid glucoside | NA |
| 423.2639 | C_24_H_38_O_6_ | M+H | 1 | M-H | 0.01 | Prenol-lipid | Ancepsenolide acetate |
| 428.3324 | C_46_H_88_NO_8_P | M+ACN+2H | 1 | NA | NA | Phospholipids | Phosphatidylcholine(20:1(11z)/18:1(9z)) |
| 428.3329 | C_48_H_88_NO_8_P | M+H+NH4 | 0 | NA | NA | Phospholipids | Phosphatidylcholine(20:1(11z)/20:3(5z,8z,11z)) |
| 445.2816 | C_41_H_75_O_13_P | M+2ACN+2H | 1 | NA | NA | Phospholipids | Phospatidylinositol(16:1(9z)/16:1(9z)) |
| 458.7769 | C_43_H_68_O13 | M+3ACN+2H | 7 | NA | NA | Terpenoid | Sanguisorbin e |
| 464.3817 | C_28_H_46_O_4_ | M+NH4 | 4 | NA | NA | Steroid | Secasterone |
| 467.2916 | C_28_H_44_O_3_ | M+H | 1 | M-H | 0.25 | Terpenoid | Australine B |
| 489.3074 | C_77_H_140_O_17_P_2_ | M+3Na | 1 | NA | NA | Prenol-lipid | Epoxy-dihydroxy-norlanostenone |
| 500.3723 | C_28_H_52_NO_7_P | M+2Na+H | 9 | NA | NA | Phospholipids | Lyso-Phosphatidylcholine(20:3(5z,8z,11z)) |
| 511.3194 | C_30_H_39_NO_5_ | M+NH4 | 5 | NA | NA | Cytotoxin | Cytochalasin npho |
| 533.3324 | C_26_H_45_NO_7_S | M+NH4 | 5 | NA | NA | Steroid | Taurocholic acid |
| 540.4029 | C_32_H_50_O_4_ | M+ACN+H | 3 | NA | NA | Terpenoid | Acetyl Ursolic Acid |
| 577.3577 | C_18_H_24_O_3_ | 2M+H | 9 | NA | NA | Steroid | Hydroxy-estradiol |
| 598.4299 | C_40_H_52_O_3_ | M+NH4 | 7 | NA | NA | Prenol-lipids | Hydroxy-diketo-b-carotene |
| 599.3706 | C_17_H_28_O_3_ | 2M+K | 0 | NA | NA | Prenol-lipids | Fauronyl acetate |
| 616.4344 | C_40_H_54_O_4_ | M+NH4 | 3 | NA | NA | Prenol-lipids | Mytiloxanthin |
| 621.3833 | C_35_H_54_N_2_O_6_ | M+Na | 7 | NA | NA | Benzoquinone | Hexaprenyl-methyl-hydroxy-methoxy-benzoquinone |
| 656.4845 | C_18_H_32_O_3_ | 2M+ACN+Na | 2 | NA | NA | Fatty-Acyl | 13s-hydroxyoctadecadienoic acid |
| 674.4945 | C_39_H_58_O_4_ | M+IsoProp+Na+H | 9 | NA | NA | Benzoquinone | Ubiquinone 6 |
| 732.535 | C_75_H_146_O_17_P_2_ | M+2ACN+2H | 1 | NA | NA | Phospholipids | unknown |
| 784.481 | C_41_H_66_O_13_ | M+NH | 3 | NA | NA | Terpenoid | Pitheduloside A |
| 828.5066 | C_43_H_70_O_14_ | M+NH4 | 5 | NA | NA | Steroid glucoside | unknown |
| 828.5068 | C_43_H_70_O_14_ | M+NH4 | 4 | NA | NA | Steroid glucoside | unknown |

1. Specific to *Acropora* sp.

| LC/MS (m/z) | Formula | Adduct | LC/MS (Δppm) | Adduct | FT-ICR (Δppm) | Chemical group | Potential Compounds |
| --- | --- | --- | --- | --- | --- | --- | --- |
| 124.9622 | NA | NA | NA | NA | NA | NA | NA |
| 132.04742 | C_6_H_12_O_2_S_2_ | M+2ACN+2H | 3 | M-H | 2 | Dithiane | 2,5-Dimethyl-1,4-dithiane-2,5-diol |
| 135.08512 | NA | NA | NA | NA | NA | NA | NA |
| 151.09592 | C_5_H_10_O_3_ | M+CH3OH+H | 3 | NA | NA | Fatty-Acyl | 5-hydroxypentanoic acid |
| 164.01872 | C_12_H_16_O_6_S | M+H+K | 1 | M-H | 0.4 | Phenol ether | {[5-(4-methoxyphenyl)-3-oxopentyl]oxy}sulfonic acid |
| 164.02232 | C_5_H_7_N_3_O | M+K | 1 | NA | NA | Diazine | 5-methylcytosine |
| 164.02622 | NA | NA | NA | NA | NA | NA | NA |
| 172.13112 | C_10_H_15_NO | M+Li | 1 | M-H | 0.1 | Benzen | Hordenine |
| 175.09422 | C_18_H_30_O_3_S | M+H+Na | 1 | M-H | 0.05 | Benzen | 2-Dodecylbenzenesulfonic acid |
| 182.1017 | C_6_H_12_O_5_ | M+NH4 | 3 | NA | NA | Sugar | Mono-hexose |
| 183.0998 | C_8_H_16_O_3_ | M+Na | 3 | NA | NA | Fatty-Acyl | hydroxyvalproic acid |
| 207.1539 | NA | NA | NA | NA | NA | NA | NA |
| 207.1561 | C_18_H_34_O_5_ | M+2ACN+2H | 1 | M-H | 0.07 | Fatty-Acyl | 9,10,13-trihome |
| 234.147 | C_19_H_40_NO_7_P | M+ACN+2H | 3 | NA | NA | Phospholipids | Lysope(0:0/14:0) |
| 234.1680 | C_36_H_72_NO_8_P | M+2H+Na | 1 | NA | NA | Phospholipids | Phosphatidylcholine(14:0/14:0) |
| 234.1682 | C_38_H_70_NO_8_P | M+3H | 2 | NA | NA | Phospholipids | Phosphoethanolamine(18:3(9z,12z,15z)/15:0) |
| 235.15795 | C_31_H_42_O_2_ | M+H+Na | 2 | NA | NA | Prenol-lipids | 3-hydroxysintaxanthin |
| 235.1614 | NA | NA | NA | NA | NA | NA | NA |
| 242.0401 | C_9_H_11_NO_4_ | M+2Na-H | 1 | NA | NA | AA derivative | Dihydroxyphenylalanine |
| 242.0471 | C_8_H_8_O_4_S | M+ACN+H | 4 | M-H | 0.02 | Arylsulfate | 4-vinylphenol sulfate |
| 242.2083 | NA | NA | NA | NA | NA | NA | NA |
| 279.1978 | C_49_H_84_O_6_ | M+3Na | 1 | NA | NA | Glycerolipids | Triglyceride |
| 295.2179 | C_10_H_13_N | 2M+H | 4 | NA | NA | Pyridines | (s)-actinidine |
| 297.2136 | C_53_H_90_O_6_ | M+3Na | 1 | NA | NA | Glycerolipids | Triglyceride |
| 298.2677 | C_37_H_70_O_5_ | M+2H | 2 | NA | NA | Glycerolipids | Diacylglycerol |
| 298.2683 | C_37_H_70_O_5_ | M+2H | 0 | NA | NA | Glycerolipids | Diacylglycerol |
| 299.1778 | C_54_H_70_MgN_4_O_6_ | M+3H | 3 | NA | NA | Tetrapyrroles | Chlorophyll d |
| 303.2255 | C_49_H_97_N_2_O_6_P | M+3Na | 0 | NA | NA | Sphingolipids | Sphingomyellin |
| 304.2265 | C_19_H_28_O_3_ | M-H2O+NH4 | 0 | M-H | 0.3 | Steroid | Hydroxytestosterone |
| 307.1983 | C_11_H_22_N_4_O_4_ | M+CH3OH+H | 2 | NA | NA | AA derivative | Lysyl-glutamine |
| 307.2040 | C_20_H_28_O | M+Na | 2 | NA | NA | Prenol-lipids | Vitamin a2 |
| 307.2202 | C_41_H_62_O_18_S | M+H+2Na | 4 | NA | NA | Terpenoid | Quillaic acid 3-[rhamnosyl-(1->3)-[galactosyl-(1->2)]-  glucuronide] |
| 317.1889 | C_18_H_30_O_2_ | M+K | 4 | NA | NA | Fatty-Acyl | Alpha-linolenic acid |
| 367.2100 | C_21_H_28_O_5_ | M+Li | 1 | M-H | 0.2 | Steroid | Aldosterone |
| 375.1911 | C_16_H_20_O_5_ | M+2ACN+H | 1 | M-H | 0.1 | Benzopyran | Coriandrone a/b |
| 375.1930 | C_16_H_20_O_5_ | M+2ACN+H | 1 | M-H | 0.1 | Benzopyran | Coriandrone a/b |
| 379.2351 | C_16_H_25_NO_4_ | M+IsoProp+Na+H | 4 | M-H | 0.15 | Phenol ether | Esmolol |
| 385.2337 | C_15_H_26_O_6_ | M+2ACN+H | 1 | M-H | 0.01 | Glycerolipids | Glycerol tributanoate |
| 393.2572 | C_41_H_70_NO_10_P | M+H+NH4 | 0 | M-H | 0.2 | Phospholipids | Phosphatidylserine |
| 401.1553 | C_17_H_21_NO_9_ | M+NH4 | 0 | M-H | 0.07 | Glucoside | 3,4,5-trihydroxy-6-{[(5E)-5-{[5-(hydroxymethyl)  furan-2-yl]methylidene}-2,3,4,5-tetrahydropyridin-  4-yl]oxy}oxane-2-carboxylic acid |
| 402.2164 | C_20_H_26_O_7_ | M+Na | 4 | M-H | 0.1 | Prenol-lipids | Niveusin c |
| 402.2233 | C_19_H_30_O_5_ | M+ACN+Na | 4 | M-H | 0.1 | Fatty-Acyl | [6]-gingerdiol 3-acetate |
| 415.2098 | C_11_H_16_O_3_ | 2M+Na | 2 | M-H | 0.05 | Fatty-Acyl | Isobutyl 2-furanpropionate |
| 416.2425 | C_24_H_32_O_6_ | M-H2O+NH4 | 0 | M-H | 0.3 | Prenol-lipids | Armillyl everninate |
| 417.1637 | C_22_H_28_O_5_ | M+2Na-H | 3 | M-H | 0.3 | Prenol-lipids | Armillasin |
| 419.1662 | C_20_H_28_O_8_ | M+Na | 4 | M-H | 0.04 | Prenol-lipids | 4,5-dihydroniveusin a |
| 425.2744 | C_42_H_71_O_10_P | M+2ACN+2H | 3 | NA | NA | Phospholipids | Phosphoglycerol(18:3(6z,9z,12z)/18:3(9z,12z,15z)) |
| 432.2363 | C_20_H_32_O_6_ | M+ACN+Na | 2 | M-H | 0.01 | Fatty-Acyl | 20-dihydroxyleukotriene b4 |
| 436.7690 | C_41_H_64_O_12_ | M+3ACN+2H | 5 | NA | NA | Prenol-lipids | 28-glucosyl-19(29)-dehydroursolic acid 3-arabinoside |

1. Specific to *Platygyra* sp.

| LC/MS (m/z) | | Formula | | Adduct | | LC/MS (Δppm) | | Adduct | | FT-ICR (Δppm) | Chemical group | Potential Compounds | |
| --- | --- | --- | --- | --- | --- | --- | --- | --- | --- | --- | --- | --- | --- |
| 438.2502 | | C_21_H_32_O_7_ | | M+ACN+H | | 4 | | M-H | | 0.01 | Prenol-lipids | Armillane | |
| 127.0870 | C_20_H_36_O_5_ | | M+2H+Na | | 2 | | M-H | | 1 | | Fatty-Acyl | Prostaglandin f1a |  |
| 174.1464 | C_9_H_18_O_3_ | | M-H2O+NH4 | | 4 | | M-H | | 0.1 | | Organic acid | 2-hydroxy pelargonic acid |  |
| 223.1561 | C_22_H_34_O_4_ | | M+2ACN+2H | | 2 | | M-H | | 0.2 | | Prenol-lipids | Dimethyl ent-16alpha-kaurane-17,19-dioate |  |
| 254.1705 | C_24_H_33_NO_3_ | | M+3ACN+2H | | 2 | | NA | | NA | | Terpenoid | (e,e,e)-sylvatine |  |
| 255.1537 | C_30_H_46_O_4_ | | M+H+K | | 5 | | NA | | NA | | Prenol-lipids | Glycyrrhetinic acid |  |
| 271.1733 | C_12_H_24_O_6_ | | M+Li | | 0 | | M-H | | 0.1 | | Glucoside | Hexyl glucoside |  |
| 271.1734 | C_13_H_20_O | | M+DMSO+H | | 3 | | M-H | | 0.05 | | Prenol-lipids | Beta-ionone |  |
| 277.1732 | C_38_H_40_O_18_ | | M+H+2Na | | 0 | | NA | | NA | | Flavonoids | Spinosin b |  |
| 301.2118 | C_13_H_25_NO_4_ | | M+ACN+H | | 1 | | M-H | | 0.03 | | Fatty-Acyl | L-hexanoylcarnitine |  |
| 352.2566 | NA | | NA | | NA | | NA | | NA | | NA | NA |  |
| 386.2437 | C_19_H_26_O_3_ | | M+IsoProp+Na+H | | 1 | | M-H | | 0.2 | | Steroid | 4-Methoxy-17beta-estradiol |  |
| 386.2430 | C_19_H_23_N_3_O_2_ | | M+IsoProp+H | | 4 | | M-H | | 0.4 | | Benzoquinone | Ergonovine |  |
| 388.2619 | C_22_H_39_NO_2_ | | M+K | | 2 | | NA | | NA | | Acylethanolamines | Dihomo-gamma-linolenoyl ethanolamide |  |
| 388.2604 | C_19_H_28_O_3_ | | M+IsoProp+Na+H | | 4 | | NA | | NA | | Steroid | Androsteron |  |
| 428.3325 | C_48_H_88_NO_8_P | | M+H+NH4 | | 1 | | NA | | NA | | Phospholipids | Phosphatidycholine(22:0/18:4(6z,9z,12z) |  |
| 442.3467 | C_16_H_2_0 | | 2M+NH4 | | 0 | | M-H | | 0.5 | | Napthatlene | 2,6-DIISOPROPYLNAPHTHALENE |  |
| 472.3558 | C_26_H_54_NO_6_P | | M-2H2O+H | | 1 | | NA | | NA | | Phospholipids | Lyso-phosphocholine(p-18:0) |  |
| 518.3234 | C_25_H_38_O_6_ | | M+IsoProp+Na+H | | 3 | | M-H | | 0.3 | | Prenol-lipids | Erinacine c |  |
| 530.3969 | C_29_H_60_NO_7_P | | M-2H2O+H | | 2 | | NA | | NA | | Phospholipids | Lyso-phosphoethanolamine(24:0/0:0) |  |
| 616.4344 | C_33_H_64_NO_8_P | | M-H2O+H | | 0 | | NA | | NA | | Phospholipids | Phosphoethanolamine(14:0/14:1(9z)) |  |
| 674.4945 | NA | | NA | | NA | | NA | | NA | | NA | NA |  |
| 732.5350 | C_75_H_146_O_17_P_2_ | | M+2ACN+2H | | 1 | | M-H | | 1 | | Phospholipids | CL(1'-[16:0/18:0],3'-[16:0/16:0]) |  |
| 742.3645 | NA | | NA | | NA | | NA | | NA | | NA | NA |  |
| 784.4810 | C_36_H_68_NO_10_P | | M+DMSO+H | | 2 | | M-H | | 2 | | Phospholipids | Phosphatidylserine(14:0/16:1(9z)) |  |
