# Supplementary material for: Coral metabolite gradients affect microbial community structures and act as a disease cue
Source: Commun Biol. 2018 Nov 5;1:184. doi: 10.1038/s42003-018-0189-1 (PMC6218554; doi:10.1038/s42003-018-0189-1)
Supplement: Supplementary file 2 — Description of additional supplementary items [file 42003_2018_189_MOESM2_ESM.docx]

**Description of Additional Supplementary Files**

**File Name**: Supplementary Data 1

**Description**: List of gradient-forming molecules shared in *Platygyra* and *Acropora* colonies or unique to each as deduced from LC-MS and FT-ICRMS data. P-values represent molecules with statistically significant relative abundance between the different distances (ANOVA and Welch’s t-test).

**File Name**: Supplementary Data 2

**Description**: List of candidate white syndrome disease indicators. P-values represent molecules with statistically significant relative abundance between healthy and diseased colonies at 0 cm (ANOVA and Welch’s t-test).
